# A Robust Miniaturized Gas Sensor for H_2_ and CO_2_ Detection Based on the 3*ω* Method

**DOI:** 10.3390/s22020485

**Published:** 2022-01-09

**Authors:** Dominik Berndt, Josef Muggli, Robert Heckel, Mohd Fuad Rahiman, Matthias Lindner, Stephan Heinrich, Heinz Plöchinger, Rupert Schreiner

**Affiliations:** 1 Faculty of Natural Sciences and Cultural Studies, OTH Regensburg, Seybothstraße 2, 93053 Regensburg, Germany; josef2.muggli@st.oth-regensburg.de (J.M.); robert.heckel@st.oth-regensburg.de (R.H.); mohd.rahiman@st.oth-regensburg.de (M.F.R.); matthias.lindner@oth-regensburg.de (M.L.); rupert.schreiner@oth-regensburg.de (R.S.); 2 System Engineering & Advanced Development, BU Sensing & Actuation, Vitesco Technologies GmbH, Siemensstraße 12, 93055 Regensburg, Germany; stephan.heinrich@vitesco.com; 3 Thyracont Vacuum Instruments GmbH, Max Emmanuel Straße 10, 94036 Passau, Germany; heinz.ploechinger@thyracont.de

**Keywords:** thermal gas sensor, 3*ω*-method, CO_2_ sensor, H_2_ sensor

## Abstract

Gas concentration monitoring is essential in industrial or life science areas in order to address safety-relevant or process-related questions. Many of the sensors used in this context are based on the principle of thermal conductivity. The **3ω**-method is a very accurate method to determine the thermal properties of materials. It has its origin in the thermal characterization of thin solid films. To date, there have been very few scientific investigations using this method to determine the thermal properties of gases and to apply it to gas measurement technology. In this article, we use two exemplary gases ($H_{2}$ and $CO_{2}$) for a systematical investigation of this method in the context of gas analysis. To perform our experiments, we use a robust, reliable sensing element that is already well established in vacuum measurement technology. This helix-shaped thin wire of tungsten exhibits high robustness against chemical and mechanical influences. Our setup features a compact measurement environment, where sensor operation and data acquisition are integrated into a single device. The experimental results show a good agreement with a simplified analytical model and FEM simulations. The sensor exhibits a lower detection limit of 0.62% in the case of $CO_{2}$, and only 0.062% in case the of $H_{2}$ at an excitation frequency of 1Hz. This is one of the lowest values reported in literature for thermal conductivity $H_{2}$ sensors.

## 1. Introduction

Gas sensors are widely used, for example in industrial, automotive, and environmental applications [1]. There are a number of different operating principles for gas sensors. In terms of hydrogen gas sensors, Huebert et al. provide a good comparison of different operating principles [2]. Metal oxide [3,4] and electrochemical sensors [5,6] are the most common types of gas sensors. Both are able to resolve very low gas concentrations and can therefore be used for the detection of gas traces. On the other hand, these sensors exhibit a measurement range limited to a few percent [2] which also applies to catalytic [7] and work function-based sensors [8,9]. For certain applications, such as human breath analysis [10], or landfill gas monitoring [11], however, a large measurement range is more important than an ultra-low resolution limit. Optical [12,13,14] and mechanical [15,16] sensors do have a large measurement range but at the expense of a slow response time. Furthermore, they are susceptible to poisoning [2]. Acoustic sensors also cover a wide measurement range while having a fast response time. However, there are still issues with long-term stability [2,17]. Thermal conductivity gas sensors represent a good compromise for these aspects as they exhibit a large measurement range, a fast response time, good long-term stability, and a resolution limit that is sufficient for a lot of use cases [1,2,10,18,19,20,21,22].

The operating principle of thermal conductivity gas sensors is based on the different thermal properties of various gases and gas compositions. A current is applied to a heating element, which induces a temperature rise and, as a consequence, a change in resistance of the heated element. This change in resistance depends on the thermal conductivity of the surrounding fluid [23]. This procedure is suitable to analyze a binary gas mixture as analytes such as hydrogen ($H_{2}$) or carbon dioxide ($CO_{2}$) have a significantly different thermal conductivity with respect to the carrier gas, which is usually nitrogen ($N_{2}$) or synthetic air.

The current can, for example, be applied in pulses. The evaluation quantity is then the height, the time constant, or the phase shift of the subsequent resistance pulse [10,21,24,25,26,27]. Besides the pulsed method, a constant-temperature operation can be established using a suitable control circuit. In this case, the temperature difference between heating element and ambient temperature is kept constant. The power required to maintain this temperature difference correlates with the thermal conductivity of the gas composition [22,28,29].

In the past decade, a frequency domain-based approach has been used more frequently. A sinusoidal current is applied to the sensor element and the thermal conductivity of the surrounding gas mixture can be derived from the amplitude of the resulting temperature oscillations. In the context of a gas sensor, this principle, known as the 3ω-method, is still subject of ongoing research with just a limited number of publications so far [30,31,32,33,34]; especially for the detection of hydrogen, there are no scientific studies yet.

The 3ω-method has the advantage that it does not depend on the thermal characteristics of the sensor and can be operated at lower temperatures [32]. In combination with a miniaturization of the sensor which has a positive effect on the power consumption of the system this technique shows a good sensitivity and resolution limit [33].

In contrast to previous studies that use micro- or nanomachined sensing elements, e.g., microbridges [33], or nanowires [34], we use a very thin tungsten wire helix. The helix is fixed on a TO socket via a micro-welding process. This robust sensor has the advantage of a simple manufacturing process without cleanroom fabrication. As the wire couples directly to the surrounding gas and is not embedded in a membrane, the signal is not smoothed by parasitic capacitances which is beneficial for dynamic operation. The sensor itself can be purchased as a Pirani gauge from the company Thyracont Vacuum Instruments, Passau, Germany. While some of the earlier works use a rather comprehensive setup consisting of power supply, source meter unit, and lock-in amplifier [30,35], our sensor is operated by a compact circuit with a digital lock-in amplifier only. This lock-in amplifier (LI) simultaneously provides the sensor power supply and the measurement data acquisition.

In the present study, we investigate the performance of a compact 3ω-based measurement system using a simple sensor design. In particular, we focus on the sensitivity and resolution limit at different excitation frequencies for the detection of $CO_{2}$ and $H_{2}$. The results are compared to other studies reporting on thermal conductivity sensors. Beyond that, we want to explore the potential and the limitations of this technique for possible applications, such as human breath analysis or food monitoring [36,37,38,39] in terms of $CO_{2}$, or fuel cell or power-to-gas applications [18,40] in terms of $H_{2}$.

## 2. Analytical Model

For a qualitative description of the system, an analytical model will be considered first. In this model, the helix is represented as a cylindrical conductor with a homogeneous temperature distribution in its centre. For this reason, the heat transport equation in cylindrical coordinates is used to describe the transient heat transport processes from the cylinder radially to the ambient. As, in our geometry, the cylinder axis corresponds to the component *z*, the initial, as well as the boundary conditions, are independent of the angular coordinate θ and the longitudinal axis *z*. Thus, the temperature *T* can be described by the radial coordinate *r* and the time *t*.

(1) $$\frac{1}{r}\frac{\partial T}{\partial r}+\frac{\partial^{2}T}{\partial r^{2}}-\frac{1}{D}\left(\frac{\partial T}{\partial t}\right)=0$$

The quantity *D* represents the thermal diffusivity

(2) $$D=\frac{\lambda }{\rho_{G}C_{G}}$$

where λ, $\rho_{G}$, and $c_{G}$ denote the thermal conductivity, the density, and the specific heat capacity of the gas, respectively. For a temperature $T=T_{0}e^{i(\omega_{T}t-\phi )}$ that is periodically modulated with $\omega_{T}$, and exhibits a phase shift of ϕ, Equation (1) is expressed as:

(3) $$\frac{1}{r}\frac{\partial T}{\partial r}+\frac{\partial^{2}T}{\partial r^{2}}=\frac{i\omega_{T}}{D}T$$

The parameter *i* denotes the imaginary unit. $T_{0}$ is the ambient temperature. Applying the boundary condition $T_{r\to \infty }=0$, a solution of Equation (3) describing the temperature *T* in relation to the radial coordinate *r* is given by Carslaw and Jaeger [41] as

(4) $$T\left(r\right)=\frac{P_{V}}{\pi l\lambda }\;\cdot \;K_{0}\left(qr\right)\;\mathrm{with}\;q=\sqrt{\frac{2i\omega_{T}}{D}}$$

where $P_{V}$ denotes the electrical power per unit volume and $K_{0}$ the zeroth-order modified Bessel function. Assuming that the length of the heating element *l* is much larger than its radius $r_{0}$, the system can be considered as two-dimensional and the power per unit volume $P_{V}$ can be described as [42]

(5) $$P_{V}=\frac{I_{\mathrm{eff}}^{2}R_{0}}{4\pi r_{0}^{2}l}$$

where $I_{\mathrm{eff}}$ is the root mean square (RMS) value of the sinusoidal input current with a frequency ω. $R_{0}$ is the resistance of the wire at room temperature. As both the positive and negative half-waves of the sinusoidal current cause heating, it applies that $\omega_{T}=2\omega$.

If we expand the Bessel function to the smallest order in the argument qr [43] and include not only the thermal properties of the fluid but also those of the heating element, we obtain the solution proposed by Yusibani et al. [44] for the temperature oscillations at the point $r=r_{0}$:

(6) $$\begin{array}{c} \Delta T_{AC}=AB\lambda \;\cdot \;\cos\left(2\omega t\right)+\left(\left(A^{2}+\frac{\pi^{2}}{16}\right)r_{0}^{2}\left(\rho c_{p}\right)\omega +\frac{\pi \lambda }{4}\right)B\;\cdot \;\sin\left(2\omega t\right) \end{array}$$

with the coefficients *A* and *B*

$$\begin{array}{c} A=(-\frac{1}{2}\ln\left(\frac{2\omega }{D}\right)-\ln\left(r_{0}\right)-\gamma +\ln\left(2\right))\\ B=\frac{I_{\mathrm{eff}}^{2}R_{0}}{2\pi l\left(r_{0}^{2}\left(\rho c_{p}\right){\omega A)}^{2}+{\left(\pi r_{0}^{2}\left(\rho c_{p}\right)\frac{1}{4}\omega +\lambda \right)}^{2}\right)} \end{array}$$

In this relationship, the parameters ρ and $c_{p}$ represent the density and the specific heat of the sensing element itself, whereas *D* and λ refer to the thermal diffusivity and conductivity of the fluid, respectively. γ denotes the Euler´s constant. As it describes the heat transport of a cylindrical conductor radially to the outside, the model represents a simplification of the real case. In addition, the temperature gradient along the longitudinal axis of the cylinder *z*, which arises due to heat losses via the suspensions, is neglected. In a later work, Yusibani et al. have developed another model including these losses, representing the temperature oscillations integrated over the length of the wire [45]. However, as we only take the temperature oscillations at one point in the center of the wire in the simulation, the simplified analytical model is a sufficient approximation for the considered problem.

## 3. FEM-Simulation

**Figure 1:**
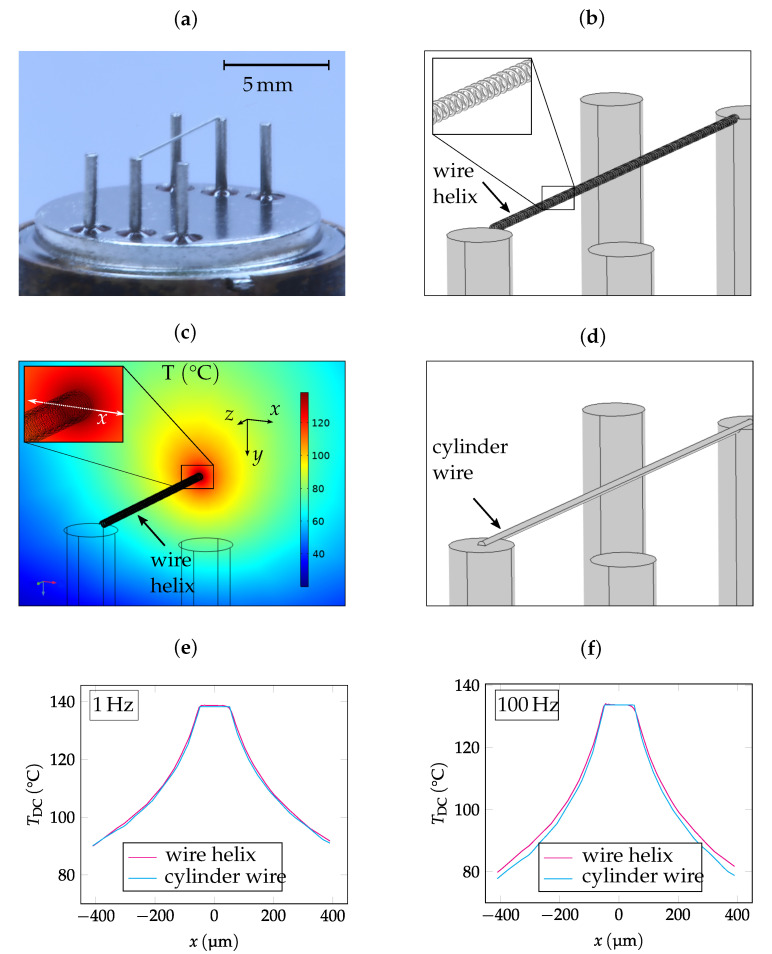
Comparison of a photograph of the helix-shaped wire sensor (**a**) with the detailed model of the sensor (**b**). The temperature distribution $T_{\mathrm{DC}}$ along the cross-sectional coordinate *x* inside the helix is homogeneous (**c**), both for low and high frequencies (**e**,**f**). Outside the wire, a temperature decay is observable. For this reason, a simplified geometry (**d**) with the same radius of 50μm can be applied for modelling. Note, that the electrical power of the simplified geometry was increased to reach the same $T_{\mathrm{DC}}$ as for the detailed geometry.

Subsequently, a finite element method (FEM) simulation was carried out using COMSOL Multiphysics. The simulation has the purpose of validating the analytical model and verifying the experimental results afterwards. In this context, a 3D model of the sensor was created which can be seen in Figure 1 in comparison to a photograph of the sensor.

The high-resolution model (see Figure 1b) depicts a detailed reconstruction of the sensor geometry. The wire has a thickness of 8 μm, is wound in a radius of 50 μm, and fixed at two PINs of a TO Socket with a distance of 2.8 mm above the bottom of the socket. The wire helix is surrounded by air. The detailed design will be explained in Section 4. The modules heat transfer in solids and fluids, as well as electric currents, were used in order to achieve Joule’s heating in and around a current-carrying conductor. Both modules are coupled using electromagnetic heating. A Dirichlet boundary condition was applied to the bottom of the TO socket. The temperature at the boundary, as well as the ambient temperature, were set to 20 °C. A sinusoidal current was applied between the ends of the wire helix. A time-dependent study was conducted for certain frequencies of the sinusoidal current. In Figure 1e,f, it can be noticed that the temperature distribution as a function of the cross-sectional coordinate *x* inside the wire helix is homogeneous after a transient time period for two exemplary frequencies (5 Hz and 100 Hz). Slight deviations occur due to heat influences from adjacent wire windings. Outside the wire helix, a temperature decay is observable. This decay corresponds to the zeroth-order modified Bessel function (see Equation (4)).

Due to the homogeneous temperature distribution, and as a time-dependent study is memory-intensive, the helix-shaped geometry in Figure 1b was substituted by a simplified model in the following calculations. In this simplified model, a cylindrical rod with the same radius of 50 μm represents the wire helix (see Figure 1d). This procedure allows the number of elements to be reduced. As a consequence, the current through the wire has to be adapted to reach the same temperature $T_{\mathrm{DC}}$ as we obtain using the detailed geometry ($T_{\mathrm{DC}}\approx 137\;{}^{\circ}C)$.

**Figure 2:**
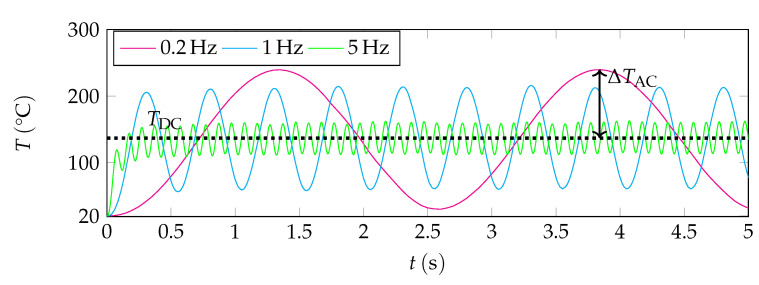
Temperature oscillations for different input current frequencies in ambient air as a function of time in the time-dependent study. The DC-component of the heater temperature $T_{\mathrm{DC}}$ is constant, whereas the amplitudes of the temperature oscillation $T_{\mathrm{AC}}$ depend on the frequency. The amplitude $\Delta T_{\mathrm{AC}}$ for a frequency of 0.2Hz is marked exemplarily. According to the explanation in Section 2, the temperature oscillations exhibit twice the frequency than the sinusoidal input current.

$T_{\mathrm{DC}}$ can also be seen in Figure 2, where the frequency dependence of the temperature oscillations is shown for three exemplary frequencies (0.2 Hz, 1 Hz, and 5 Hz). Here, the temperature of the sensor measured at a central point directly on the cylinder surface is plotted versus time. As it can be seen from Figure 2, the temperature in this point oscillates around a certain level $T_{\mathrm{DC}}$. The amplitude of these oscillations is marked with $\Delta T_{\mathrm{AC}}$. Due to the thermal capacitance, respectively the low-pass behaviour of the sensor element, the amplitude of the temperature oscillations $\Delta T_{\mathrm{AC}}$ experiences an attenuation at higher frequencies. The RMS values of these amplitudes $\Delta T_{\mathrm{AC}}$ for different frequencies are compared to the results of the analytical model (see Equation (6)) and the experimental data in Section 5.

## 4. Measurement Setup

As mentioned before, the helix-shaped sensor element is formed by a thin tungsten wire with a diameter of 8 μm and winding radius of 50 μm. It is fixed by a micro-welding process at two PINs of a TO socket. The distance of these two PINs is 7.62 mm, which defines the length of the sensor element. By using a helix-shaped geometry, the gas-coupling surface area can be maximized while maintaining the length of the sensor element for a compact design [46]. This results in a surface area that is similar to most micro-hotplate designs [47]. At the same time, however, mechanical stability is increased as the sensor element is not under tension, and is less sensitive to breakage due to vibrations [48] in contrast to conventional membranes or nanowires. Tungsten is characterized by its robustness, especially by the fact that it has the highest melting point and one of the highest tensile strengths of all metals. Additionally, its chemical reactivity and oxidation rate are very low at temperatures below 200 °C [49]. A picture of the sensor can be seen in Figure 1a.

The sensor was encapsulated in an airtight module. This ensures that the gas concentration does not change during the measurement. The electrical contacts were isolated by a sealing material and led to the periphery. The module was screwed in a measuring rail together with a $CO_{2}$ reference sensor from Sensirion, Stafa, Switzerland (STC31). The STC31 serves as a benchmark in terms of response time. Furthermore, it is useful for a cross-check of the desired gas concentration. Gas lines, which are connected to a system of gas cylinders, are adapted at one end of the rail. In between, mass flow controllers (MFCs) regulate the gas flow and thus set the desired gas concentration. Each analyte is diluted with $N_{2}$, as this carrier gas has a very similar thermal conductivity to synthetic air. A gas mixture would unnecessarily increase the measurement uncertainty in the gas concentration. Beyond that, the risk of combustion or explosion is eliminated in the absence of oxygen as we reach a critical value of hydrogen (4%). Valves are installed after the MFCs, as well as before and after the measuring rail. These valves are closed during static measurements in order to prevent the influence of convection. In this way, very low hydrogen or carbon dioxide concentrations can be provided. Nitrogen serves as the dilutant gas in both cases. The other end of the rail leads to the gas extraction.

The sensor is connected to a circuit board, which in turn is connected to a digital LI (HF2LI from Zurich Instruments, Zurich, Switzerland). This LI has the advantage of an integrated 14-bit analog-to-digital converter (ADC). The LI is connected to a computer via USB and controlled by a Matlab code enabling a very fast integrated data acquisition. Another advantage of the HF2LI is its analog voltage output, which makes it possible to output a sinusoidal voltage with a certain frequency. The internal signal generation then automatically locks the lock-in amplifier to this frequency (or the third harmonic of this frequency, respectively). Otherwise, the process of frequency locking can be very time-consuming, particularly at low frequencies. The printed circuit board consists of two branches. The first branch is used to provide the signal as it converts the sinusoidal voltage signal provided by the LI into a current signal. This current signal is passed through the sensor and a reference resistor connected in series and then into the second branch. There, the voltage across the sensor and reference resistor is fed into two instrumentation amplifiers of type LT1167. The two voltages can be matched to each other by employing different gain factors, even if the resistance values of the sensor and reference resistor differ from each other.

**Figure 3:**
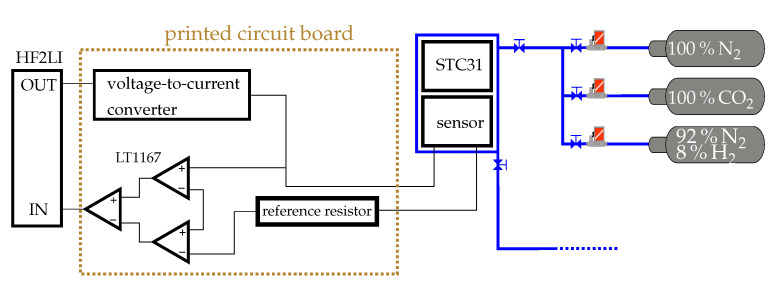
Schematic overview of the measurement setup: a sinusoidal voltage is provided by the lock-in amplifier (HF2LI) and transferred to a current by a voltage-to-current converter. From there the current is fed into the sensor and a reference resistor in line. The sensor is located in a gas pipeline, where a reference sensor (STC31 from Sensirion) is also present. The desired gas concentration is provided by a system of MFCs. The sensor signal is processed by instrumentation amplifiers (LT1167) and subsequently read out and digitized by the HF2LI.

In contrast to previous studies, e.g., by Kommandur et al. [33] or Schiffres et al. [30], where this matched voltage is directly fed into the differential input of the lock-in amplifier, we have experienced that an external component, i.e., a third instrumentation amplifier has a better common-mode rejection ratio, which is above 90 dB. This is obvious as this component is designed for low frequencies. Commercially available LIs are much lower; the HF2LI, for example, is at 80 dB. Therefore, for low-frequency applications below 1 kHz, we suggest the use of an external instrumentation amplifier instead of the differential input of the LI and to feed the subtracted signal into the non-differential input of the LI. A complete overview of the measurement setup can be found in Figure 3.

## 5. Results

### 5.1. Validation of the Experimental Results

First of all, a measurement in ambient air was performed and compared with the analytical results from Section 2 and simulation results from Section 3 in order to validate the measurement results and the functionality of the measurement setup. For this reason, $V_{3\omega }$ was measured at 50 different frequencies from 1 to 1000 Hz. In this range, the frequency values were selected with an equidistant spacing on a logarithmically scaled abscissa. For the experimental data, the amplitude of the temperature oscillations was calculated from $V_{3\omega }$ using the relationship of Mart et al. [50]:

(7) $$\Delta T_{\mathrm{AC}}=\frac{2V_{3\omega }}{\alpha RI}$$

where *I* is the RMS value of the current applied to the sensor, *R* the resistance of the sensor measured at room temperature, and α the temperature coefficient of resistance of the heating element, which was obtained in experiments as $4.1\times 10^{-3}$ K${}^{-1}$.

The analytical data were obtained for different frequencies using Equation (6). Based on a wire diameter of 8 μm and a winding radius of 50 μm, the thermal quantities ρ and $c_{p}$ were calculated in proportion from tungsten and air. The values used for the sensor-specific thermal and electrical quantities are illustrated in Table 1.

**Figure 4:**
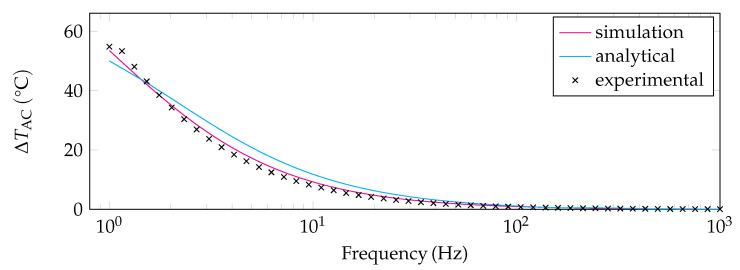
Comparison of experimental data to the simulation data and the analytical results in case of ambient air.

The simulation data of the temperature oscillations were also obtained at different frequencies. The amplitudes $\Delta T_{\mathrm{AC}}$ were extracted for the second half of the time-dependent studies (see Figure 2) and the corresponding RMS values were calculated. This ensures that the amplitude does not change anymore. A power-curve fit of the simulation data, the analytical solution, and the results of the measurements are visualized in Figure 4. The results of the analytical solution differ slightly from those obtained in the simulation and experiment. This might be due to deviations of the model from the real case, e.g., different heat capacities of the simplified geometry (analytical study, numerical study) and the real geometry (experimental study), and heat loss via suspensions (see Section 2). In addition, the influence of convection which can be seen in Figure 1c is not entirely negligible. Furthermore, the PINs where the wire helix is fixed have a large heat capacity which is not considered in the analytical study at all. In general, however, the basic trend of the analytical solution is well represented and thus suitable for the qualitative description of the effects. However, most of the effects that are missing in the analytical study are taken into account in the simulation. Therefore, the results of the numerical study are in better agreement with the experiment. As the distance between the sensor element and heat sink is rather high (2.8 mm), the thermal penetration depth is supposed to have almost no influence on the analytical and numerical studies—only the deviation towards very low frequencies between simulation and experiment might be associated with a rising influence of the thermal penetration depth.

### 5.2. CO_2_ Measurements

After the measurement results in air were successfully validated using the simulation data and the analytical study, measurements of $CO_{2}$ in $N_{2}$ were performed. In this context, six different concentrations ranging from 0.13% to 5.2% of $CO_{2}$ diluted in $N_{2}$ were considered. Due to the low concentrations of $CO_{2}$, there was a slight deviation between the $CO_{2}$ concentration set by the MFCs and the values measured by the STC31 reference sensor. As, for the STC, the accuracy of the measured value is specified as ±3% [51], the STC31 values were used as a reference here. Additionally, a measurement for 100% $CO_{2}$ was performed to show that a measuring range up to 100% is feasible. For all concentrations, four different output quantities were considered: the amplitude $V_{3\omega }$, the in-phase *X* and the out-of-phase component *Y* of the 3ω-signal, as well as the phase shift *P* between the temperature oscillations and the sinusoidal current signal. $V_{3\omega }$ and *P* can be calculated manually by applying Equation (8):

(8) $$V_{3\omega }=\sqrt{X^{2}+Y^{2}}\;P=arctan2\left(Y,X\right)$$

**Figure 5:**
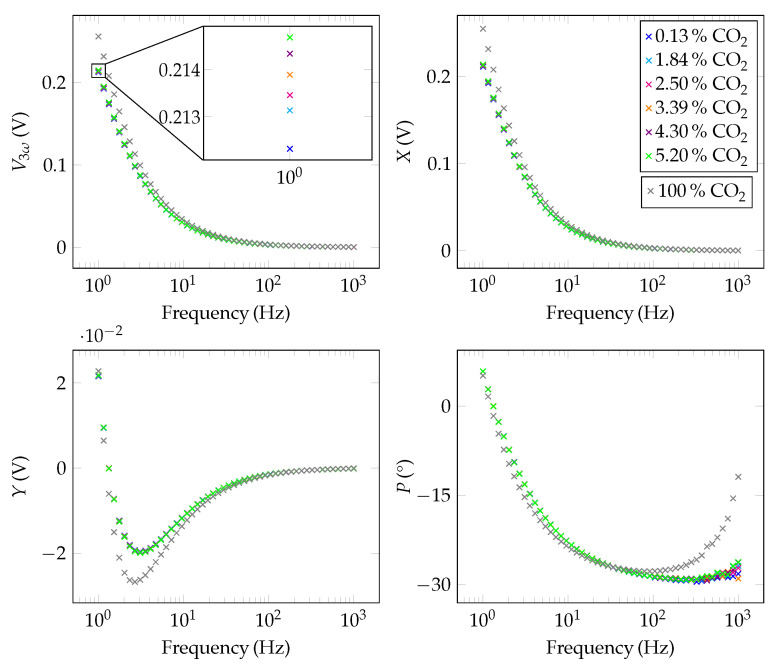
Raw 3-Omega signals (amplitude ($V_{3\omega }$), in-phase component (*X*), out-of-phase component (*Y*) and phase (*P*)) for different concentrations of $CO_{2}$ in $N_{2}$.

In our setup, the digital lock-in amplifier does this automatically [52]. These parameters were recorded at 50 different frequencies from 1 Hz to 1000 Hz. In this context, we first waited for 10 oscillations to ensure that the system has been stabilized; after that, the values for $V_{3\omega }$, *X*, *Y*, and *P* were then averaged over 20 oscillations with a 30 kS s^−1^ sampling rate for each frequency. The results can be seen in Figure 5. The lower the excitation frequency is set, the better is the discriminability to different gas mixtures. This is especially recognizable for the amplitude, the in-phase component, and the out-of-phase component. As *X* and *Y* move to zero in the high-frequency range, errors in the phase-lag *P* become more noticeable in this range and distort the measurement. These uncertainties mainly arise from time lags of the electronic components used between signal provision and data acquisition, e.g., voltage-to-current converter or instrumentation amplifiers.

**Figure 6:**
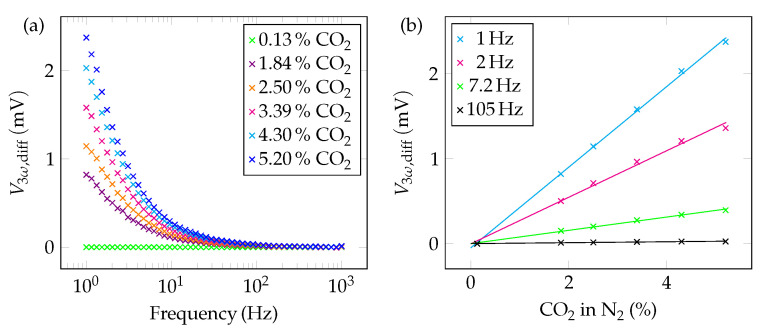
(**a**) Differential sensor response for carbon dioxide with respect to 100% $N_{2}$ ($\hat{=}0\%\;$ CO_2_) as a function of the frequency. In the low-frequency range (from 1 Hz to 10 Hz) the values are well distinguishable. (**b**) Differential sensor output as a function of the CO_2_ concentration in the single-digit percentage range plotted for on the data point at 0% CO_2_. The slope of the linear regression curve gives a hint to the sensitivity of the setup, i.e. the higher the slope, the higher the sensitivity.

As it can be seen from Figure 5, the curves for the individual concentrations are hardly separatable. For better visualization, a differential illustration is chosen. Besides *X*, the amount $V_{3\omega }$ is best suited to distinguish the individual curves from each other, as it contains *X* as well as *Y*. Note that in Figure 5 the scaling is different for *Y*. For this reason, the differential sensor signal $V_{3\omega ,diff}$ for various $CO_{2}$ concentrations is plotted vs. the frequency in Figure 6a. The single concentration curves can be distinguished well in a frequency range from 1 Hz to 10 Hz. This behaviour can also be seen in Figure 6b. Here, a linear rise of the sensor signal with increasing $CO_{2}$ concentration can be observed. The slope of the linear graph is positive as $CO_{2}$ has a lower thermal conductivity than $N_{2}$. The sensitivity of the sensor system can be obtained directly from this plot. If an excitation frequency of 1 Hz is selected, the slope of the straight line is high and a sensitivity of 0.47 mV%^−1^ is obtained. The higher the frequency, the worse the sensitivity. At 105 Hz, for example, the sensitivity only reaches a value of 5.46 μV%^−1^.

### 5.3. H_2_ Measurements

**Figure 7:**
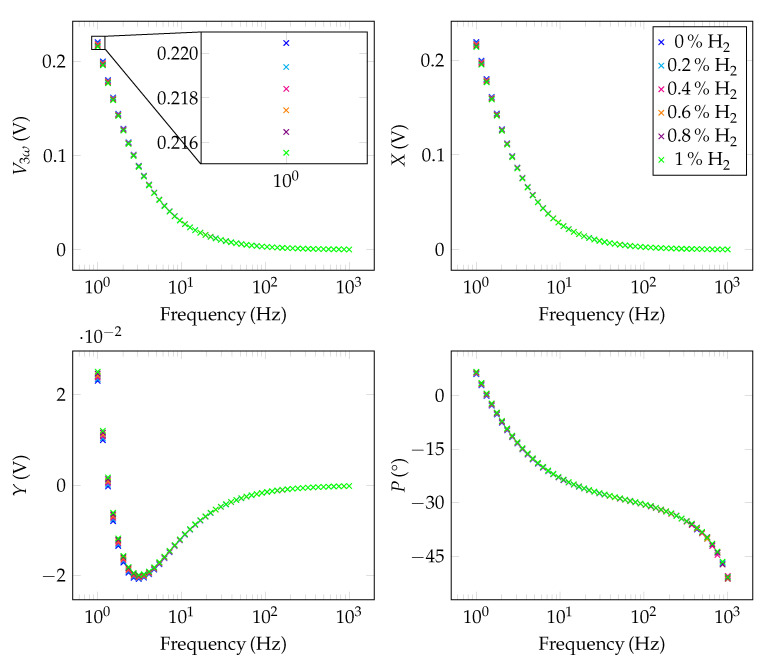
Raw 3-Omega signals (amplitude ($V_{3\omega }$), in-phase component (*X*), out-of-phase component (*Y*) and phase (*P*)) for different concentrations of $H_{2}$ in $N_{2}$.

As hydrogen gas has a thermal conductivity seven times higher than that of nitrogen, much smaller percentage steps were selected than for the $CO_{2}$ measurements in Section 5.2. Therefore, the sensor was exposed to five different concentrations (0.2%, 0.4%, 0.6%, 0.8%, and 1.0%). These low concentrations can be reliably controlled by the MFCs as $H_{2}$ is already diluted to only 8% in $N_{2}$ in the gas cylinder (see Figure 3). The results were again compared with a measurement of 100% of $N_{2}$ and depicted in Figure 7. The course of the signal is basically the same as in Figure 5. Differences can again only be accurately detected in the differential plot.

**Figure 8:**
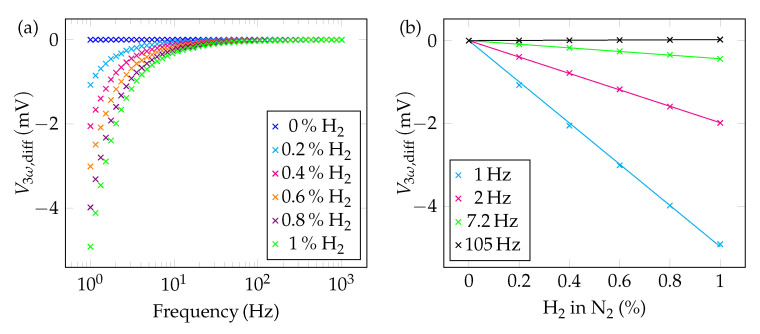
(**a**) Differential sensor response for hydrogen with respect to 100% $N_{2}$ ($\hat{=}0\%\;$ H_2_) as a function of the frequency. Although the analyzed concentrations are much smaller than those of the CO_2_ measurements, the differential signals are larger, which is due to the higher thermal conductivity difference of H_2_ and N_2_ compared to CO_2_ and N_2_ (**b**) Differential sensor signal as a function of the H_2_ concentration plotted for different frequencies. All data points are differentiated on the data point at 0% H_2_.

In this context, Figure 8a shows the differential sensor response with respect to 100% of nitrogen. The measurement points are equidistantly spaced at all frequencies. This is also shown in Figure 8b, where, similar to the $CO_{2}$ measurements, the sensor response was plotted against the $H_{2}$ concentration. Here, the straight lines show a negative slope. This is due to the fact that the thermal conductivity of hydrogen is higher than that of nitrogen. Therefore, compared to 100% $N_{2}$, the amplitude of $V_{3\omega }$ decreases. Again, if we look at the sensitivity for 1 Hz and 105 Hz, we find values of 4.97 mV%^−1^ and 23.3 μV%^−1^, respectively, from the magnitude of the slope.

### 5.4. Time-Dependent Characteristics

**Figure 9:**
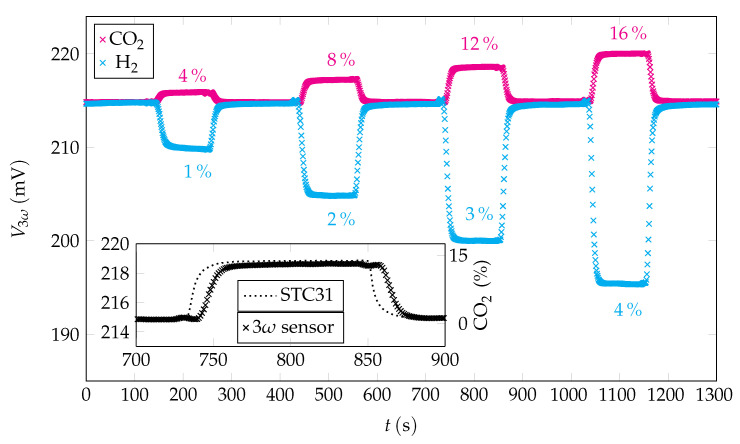
Different $H_{2}$ and $CO_{2}$ concentrations vs. time. Due to the lower limit of the MFCs, the concentrations had to be increased with respect to the concentrations measured before. $CO_{2}$ causes a reduction in the thermal conductivity and therefore results in a higher output signal, whereas $H_{2}$ has the contrary behaviour. The inbox plot shows an exemplary comparison of the signal of our sensor to the output of an STC31 reference sensor at 12% $CO_{2}$ in $N_{2}$. The response time of the 3ω sensor is about 10 s delayed with respect to the reference sensor.

Figure 9 shows a time-dependent study of different hydrogen and carbon dioxide concentrations in nitrogen over a period of about 23 min. The amplitude of $V_{3\omega }$ was one after another recorded for $CO_{2}$ concentrations of 4%, 8%, 12%, and 16% and $H_{2}$ concentrations of 1%, 2%, 3%, and 4%. As a low gas flow of 100 mL min${}^{-1}$ had to be set for a time-dependent study of different gas mixtures, the percentage values were selected higher than in the previous studies (see Figure 5 and Figure 7). In between each period with specific concentrations of $H_{2}$, or $CO_{2}$ respectively, the measuring rail was exposed to $N_{2}$ with the same flow rate. Before the measurement was recorded, this procedure was first tested in terms of stability and reproducibility in several runs, which is Vitesco’s standard procedure for gas sensor tests. Within a one-second timestamp, 20 values are determined over 100 ms, which are then averaged to one value. This ensures an accurate time synchronization of the STC31 reference sensor. As also shown in Figure 6 and Figure 8, the sensor response increases with growing $CO_{2}$ concentration, whereas a linear decrease in the signal can be observed for $H_{2}$. In addition to our sensor, a reference sensor STC31 from Sensirion was integrated into the measuring rail. The operation mode of this sensor is also based on thermal conductivity. In the inbox-plot of Figure 9, we compared both sensors in terms of response and recovery time. Our sensor was operated at a frequency of 1 Hz. In this context, we obtain response times of 18 s for the STC31, and 28 s for our sensor. As the response time in the Sensirion data-sheet is specified as being less than one second [51], it appears that the measured response time of the STC31 correlates with the time it takes for the entire gas mixture to reach the sensor element. The recovery times are determined to 14 s for the STC31, and 23 s for our sensor.

## 6. Discussion

From the sensitivity determinations in Section 5.2 and Section 5.3, we are able to calculate the minimum resolution limit of our system. Based on an input voltage range of ± 2 V and an integrated 14 bit ADC, we see a minimum detectable concentration of 0.62% $CO_{2}$ as well as 0.062% $H_{2}$ at a frequency of 1 Hz following the procedure of Kommandur et al. [33]. The sensitivity values and resolution limits are summarized in Table 2.

At low frequencies, the temperature oscillations are rather slow and the operation can be approximated as quasi-static. The thermal conductivity λ, therefore, has the strongest influence in this frequency range. The sensitivity, as well as the resolution limit, to $H_{2}$ are significantly better than to $CO_{2}$ at a frequency of 1 Hz. This is as the thermal conductivity of $H_{2}$ differs more significantly from $N_{2}$ than that of $CO_{2}$. At higher frequencies, the thermal diffusivity *D* becomes more important as this parameter describes how fast heat can be transported. *D* does not only depend on the thermal conductivity, but also on the specific heat capacity and the density of the gas (see Equation (2)). λ and *D* are shown for $H_{2}$, $CO_{2}$, and $N_{2}$ in Table 3. There, it can be seen that the ratios of $\lambda_{H_{2}}$ and $\lambda_{N_{2}}$, as well as $D_{H_{2}}$ and $D_{N_{2}}$, respectively, correspond to a factor of ≈7 in both cases. On the other hand, the ratio of $\lambda_{N_{2}}$ and $\lambda_{CO_{2}}$ exhibits a factor of 1.6, whereas the ratio of $D_{N_{2}}$ and $D_{CO_{2}}$ gives a factor that is greater than 2. Thus, at a frequency of 105 Hz, the sensitivity and resolution limit to $H_{2}$ are still better than to $CO_{2}$, but the proportion becomes smaller due to the increasing influence of *D* [53], which is illustrated in Table 2.

As output quantities and measurement setups differ from study to study, it is difficult to compare the sensitivity values of various studies. For this reason, the resolution limits of different works are compared with the resolution limit of this study at a frequency of 1 Hz.

In the case of $CO_{2}$, the studies of Cai et al. [24], and Kliche et al. [10], which are both based on time-domain readout circuits, achieve a resolution limit of 94 ppm, and 2000 ppm, respectively. The work of Kommandur et al. [32] is also based on the 3ω-method and obtains a resolution limit of 4970 ppm, which is comparable to the resolution limit of our study. Thus, this technique is not yet suitable for applications, where it is necessary to resolve lower $CO_{2}$ concentrations, e.g., for air-quality monitoring [54]. However, there might be other use cases such as human breath analysis [10] or landfill gas monitoring [11]. In the case of $H_{2}$, we can use our own study as a benchmark. There, we compared steady-state and transient (pulsed) readout-circuits and obtained a minimum resolution of 2000 ppm [21]. Struk et al. use a similar procedure and obtain a resolution limit of 1037 ppm [26]. Simon et al., who use a constant temperature circuit, achieved a value of 2000 ppm, too [22]. Van Vroonhoven et al. achieve a resolution of 600 ppm to 700 ppm with a phase-domain readout circuit [27]. In our setup, we were able to achieve a resolution limit of 620 ppm. This is a very good performance, especially for a sensor that, unlike all the studies referenced above, does not have to be manufactured by complex cleanroom fabrication and is embedded in a compact measuring environment. There are no other studies based on the 3ω-method for the detection of hydrogen, so far. The resolution limit achieved in this study is sufficient to cover a wide range of use cases from leakage detection in automotive to fuel cell applications [22]. It appears that particularly dynamic or pulsed methods seem to be advantageous for the detection of $CO_{2}$. This observation is based on the fact that Cai et al. [24] and Kliche et al. [10] were able to resolve very low concentrations using dynamic methods, whereas the resolution limit for 3ω-based studies [32,33] was significantly higher. In the case of $H_{2}$, a (quasi)-static operation, such as low-frequency 3ω-method or steady-state operation, might be better. This hypothesis is supported by a comparison with our previous study [21]. In the present study, we obtained a better resolution limit for $H_{2}$ than in our previous study, where a MEMS-based sensing element was operated by a pulsed method.

In addition to the influence of the excitation frequency, Kommandur et al. also point out that the sensitivity can be further improved with a higher operating current [32]. However, this would be at the expense of higher power consumption. For example, a current of 30 mA modulated with a frequency of 1 Hz results in a power consumption of 9 mW per measuring cycle.

The difference in response and recovery times between our sensor and the STC31, which is about 10 s, mainly arises from the more complex post-processing of the data, as well as the high settling times for large low-pass orders and time constants. These are required in order to reach a low cut-off frequency [52]. As our focus in the present work was on a high sensitivity, as well as a low resolution limit, rather time-consuming values were chosen here, i.e., an eighth-order low-pass filter and a time constant of 1.63 ms. A further reduction in the excitation frequency is possible, and presumably would lead to an even lower resolution limit. However, this would be at the expense of a further enlargement in response time as the signal delay is further increased for higher-order low-pass filters. It is therefore important to find a balance between response time and sensitivity. At the same time, however, this holds the potential for a selective mode of operation. Depending on the desired accuracy, an operation of the sensor at different frequencies is possible. For a coarse resolution, a frequency of about 10 Hz might be sufficient. As, in this case, lower-order low-pass filters could be chosen, this has the advantage of a lower signal delay, and a faster response time. On the other hand—for a small concentration of the analyte—an operation at low frequency gives a higher resolution and sensitivity.

As mentioned in the introduction, the sensor has the advantage of a fast manufacturing process. However, this advantage is only applicable to small quantities. For larger quantities, a serial procedure such as wafer-level manufacturing is faster and more cost-effective. We also stated that the sensor geometry is beneficial for dynamic operation, as the sensor element is not embedded in an additional material. However, as it can be seen in Figure 5 and Figure 7, the sensor performance is limited at higher frequencies. Compared to MEMS-sensors, our sensor has a larger thermal capacity and therefore is underperforming in terms of frequency response.

Effects such as gas turbulence and convection could be significantly reduced by a diffusion cap. In Figure 9, the influence of the temperature can also be observed well. There, it can be seen that $V_{3\omega }$ shows a slight increase during the entire measuring time in the case of $CO_{2}$, although the same gas composition is present at the beginning, and the end of the measurement. This rise of $V_{3\omega }$ is due to the poor heat dissipation through $CO_{2}$. However, the effect of temperature can be canceled out in a temperature-dependent calibration. In addition to the temperature, the influence of humidity, as well as pressure, could be controlled by a reference sensor such as the Bosch BME680, for example.

## 7. Conclusions

In this work, we presented a compact 3ω measurement system using a robust wire sensor element. A simplified numerical model exhibits a good agreement with an analytical study and the experiments. Different concentrations of $CO_{2}$ and $H_{2}$ could be successfully detected in $N_{2}$, both in the static and in the time-dependent case. A measuring range up to 100% could be covered in the case of $CO_{2}$. A similar measurement of 100% $H_{2}$ was not possible due to safety reasons. The sensitivity and the resolution limit were calculated to both $CO_{2}$ and $H_{2}$. It can be noticed that both the sensitivity and resolution limit are significantly better in the case of $H_{2}$ than in the case of $CO_{2}$. Compared to other studies which also use thermal sensors, the resolution limit to $CO_{2}$ is definitely improvable and cannot compete with other thermal sensors yet. In the case of $H_{2}$, however, the resolution limit achieved is among the lowest values reported in the literature. In addition, this was the first 3ω-study of $H_{2}$. There are studies of helium (He) [31,32,33], but they are not exactly comparable to $H_{2}$. We have thus performed an additional study of He, which shows significant differences in the sensitivities and resolution limits compared to $H_{2}$ (see Supplementary Material). For future work, it might be interesting that the HF2LI used in this study also has the advantage of a multi-frequency option where multiple frequencies (e.g., a low, a medium, and a high frequency) can be demodulated simultaneously [55]. This would speed up the data acquisition process and improve the response time. For a real use case, the setup used in this study is still too extensive. However, it would be conceivable to use a customized solution due to the low-frequency bandwidth. For example, there is software [56] or CMOS-based [57] lock-in amplifier solutions. In this context, de Graaf et al. report a LI built upon a single circuit board for low-frequency sensor applications [58].

## Figures and Tables

**Table 1:** Thermal and electrical values used for the analytical calculations using Equation (6). The electrical parameters $I_{\mathrm{eff}}$ and *R* were taken from the simulation, and the thermal quantities ρ and $c_{p}$ were calculated proportionally for tungsten and air using literature values.

Quantity	$I_{\mathrm{eff}}\;\left(A\right)$	$R_{0}\;\left(\Omega \right)$	l(mm)	$r_{0}\;\left(m\right)$	$\lambda_{f}\;\left(W\;m^{-1}\;m^{-1}\right)$	$D_{f}\;\left(m^{2}\;s^{-1}\right)$	$\rho \;(\mathrm{kg}\;m^{-3})$	$c_{p}\;\left(J\;{\mathrm{kg}}^{-1}\;K^{-1}\right)$
Value	1.37	0.06	7.62	50e06	0.02587	1.9e−05	6.52e03	751.5

**Table 2:** Overview of the sensitivities as well as resolution limits at 1 Hz and 105 Hz, respectively, for both $CO_{2}$ and $H_{2}$ measurements. The ratios of both quantities become smaller as the frequency increases. Note that the ratio was inverted in case of the resolution limit in order to find ratios greater than 1.

	Sensitivity (1 Hz)	Sensitivity (105 Hz)	Res. Limit (1 Hz)	Res. Limit (105 Hz)
$CO_{2}$	0.47mV%^−1^	5.46μV%^−1^	0.62%	44.86%
$H_{2}$	4.97mV%^−1^	23.3μV%^−1^	0.062%	10.13%
($H_{2}$/$CO_{2}$)	10.57	4.27		
($CO_{2}$/$H_{2}$)			10.0	4.43

**Table 3:** Overview of the thermal conductivity and thermal diffusivity for $CO_{2}$, $H_{2}$, and $N_{2}$ at 25 °C.

	$\lambda (\mathrm{mW}\;m^{-1}\;K^{-1})$	$D\;(10^{-6}\;m^{2}/s)$
$N_{2}$	25.83	21.95
$CO_{2}$	16.24	10.58
$H_{2}$	180.7	155.4

## Data Availability

The data presented in this study are available on request from the corresponding author.

## Supplementary Materials

The following are available at https://www.mdpi.com/article/10.3390/s22020485, 3ω-study of Helium.

## Abbreviations

The following abbreviations are used in this manuscript:

LI	Lock-In Amplifier
$CO_{2}$	Carbon dioxide
$H_{2}$	Hydrogen
$N_{2}$	Nitrogen
He	Helium
RMS	Root mean square
FEM	Finite element method
MFCs	Mass flow controllers
ADC	Analog-to-digital converter
