# Supplementary material for: A Robust Miniaturized Gas Sensor for H2 and CO2 Detection Based on the 3ω Method
Source: Sensors (Basel). 2022 Jan 9;22(2):485. doi: 10.3390/s22020485 (PMC8780541; doi:10.3390/s22020485)
Supplement: Supplementary file 1 [file sensors-22-00485-s001.zip › sensors-1446780-supplementary.pdf]

Supplementary material to the article „A Robust Miniaturized Gas Sensor for H2 and CO2 Detection Based on the 3ω Method“

In addition to the studies on hydrogen and carbon dioxide, a study of He was also carried out. The measured values are shown in Fig. S1. Due to the larger concentrations compared to H2, the individual concentrations can be easily distinguished even without a differential plot. Helium has a comparable thermal conductivity to hydrogen, so one would expect the measured values to be very similar to those of H2. However, if we look at the sensitivity, there are significant differences, which were determined from Fig. S2. At 1Hz we get a sensitivity to helium of 3.19mV/% (compared to H2: 4.97mV/%). At 105Hz we get a value of 18µV/%. (compared to H2: 23µV/%). The difference between He and H2 is also recognizable when considering the resolution limit. For 1Hz we got a resolution limit of 0.17% (compared to H2: 0.062%) and for 105Hz a resolution limit of 13.62% (compared to H2: 10.13%). These values suggest that it does matter whether helium is used instead of hydrogen in a study for a thermal hydrogen sensor.

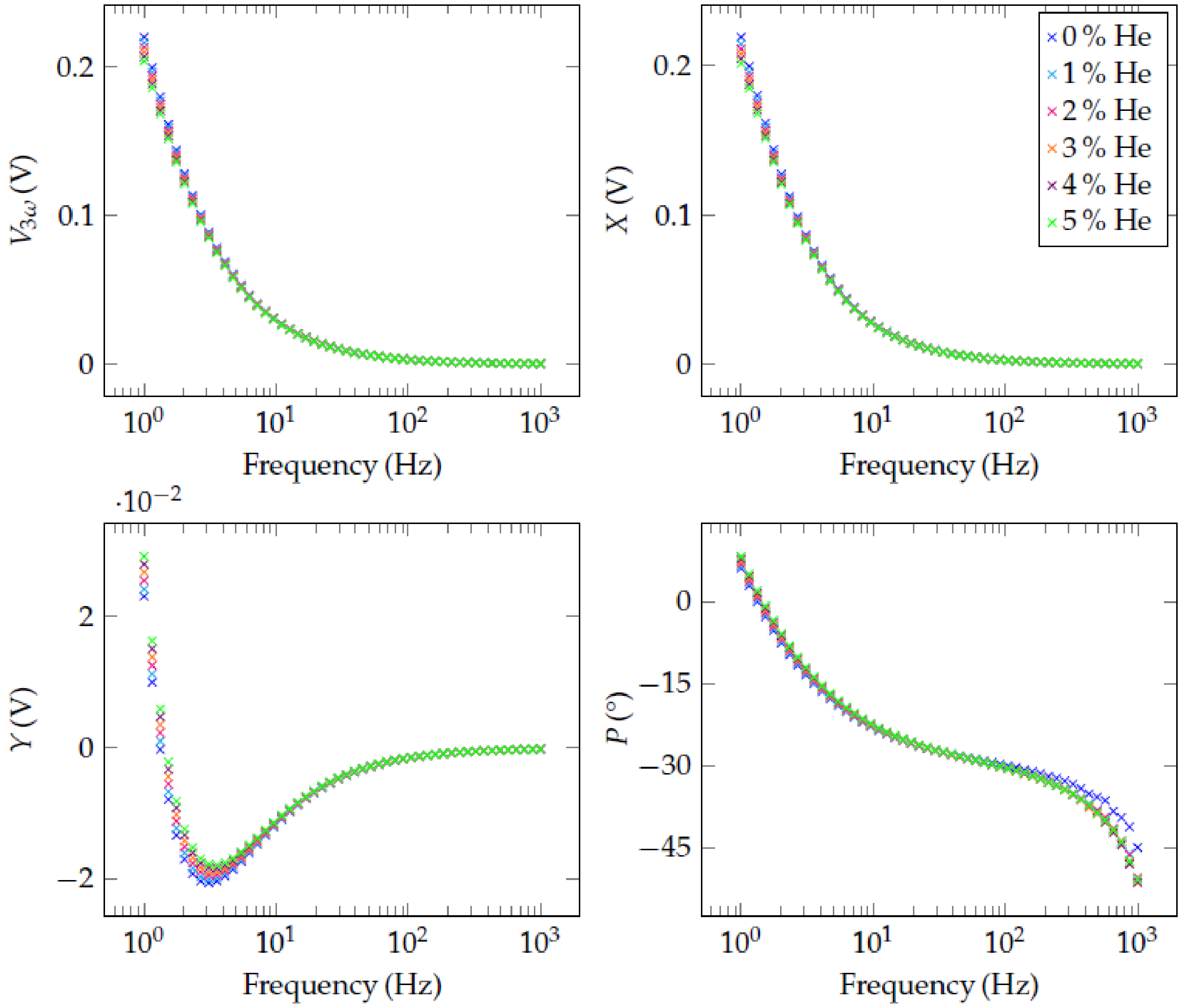

Figure S1. Raw 3-Omega signals (amplitude ($V_{3\omega}$), in-phase component (X), out-of-phase component (Y) and phase (P)) for different concentrations of He in N_2.

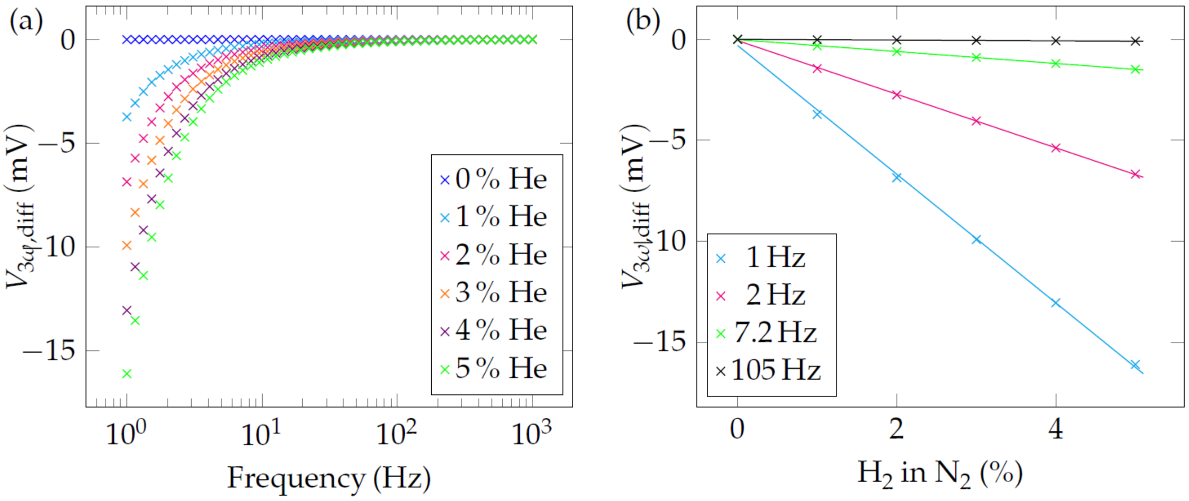

Figure S2. (a) Differential sensor response for helium with respect to 100 % N_2 ($\widehat{=}$ 0 % He) as a function of the frequency. (b) Differential sensor signal as a function of the He concentration in the single-digit percentage range plotted for different frequencies. All data points are differentiated on the data point at 0 % He.
